# Anemia in heart failure: still an unsolved enigma

**DOI:** 10.1186/s43044-021-00200-6

**Published:** 2021-08-28

**Authors:** Yash Paul Sharma, Navjyot Kaur, Ganesh Kasinadhuni, Akash Batta, Pulkit Chhabra, Samman Verma, Prashant Panda

**Affiliations:** Department of Cardiology, Post Graduate Institute of Medical Education and Research (PGIMER) Chandigarh, Sector-12, Chandigarh, India

**Keywords:** Anemia, Erythropoietin, Erythropoietin-stimulating agents, Heart failure, Iron deficiency, Parenteral iron

## Abstract

**Background:**

Anemia affects one-third of heart failure patients and is associated with increased morbidity and mortality. Despite being one of the commonest comorbidities associated with heart failure, there is a significant knowledge gap about management of anemia in the setting of heart failure due to conflicting evidence from recent trials.

**Main body:**

The etiology of anemia in heart failure is multifactorial, with absolute and functional iron deficiency, decreased erythropoietin levels and erythropoietin resistance, inflammatory state and heart failure medications being the important causative factors. Anemia adversely affects the already compromised hemodynamics in heart failure, besides being commonly associated with more comorbidities and more severe disease. Though low hemoglobin levels are associated with poor outcomes, the correction of anemia has not been consistently associated with improved outcomes. Parenteral iron improves the functional capacity in iron deficient heart failure patients, the effects are independent of hemoglobin levels, and also the evidence on hard clinical outcomes is yet to be ascertained.

**Conclusion:**

Despite all the research, anemia in heart failure remains an enigma. Perhaps, anemia is a marker of severe disease, rather than the cause of poor outcome in these patients. In this review, we discuss the current understanding of anemia in heart failure, its management and the newer therapies being studied.

## Background

Heart failure (HF) is a clinical syndrome characterized by inability of heart to perform circulatory function efficiently due to structural and/or functional abnormalities. It continues to be an important global health issue with an estimated worldwide prevalence of more than 37.7 million [1]. With an epidemic of coronary artery disease, diabetes mellitus and other life style diseases, it is estimated that globally the number of HF patients would increase by 25% by the year 2030 [2]. Despite best of medical and device therapies, the mortality rate of HF patients is 50% at 5 years of diagnosis [3], which is more than that of breast, prostate and colon cancer [4].

One-third of HF patients are anemic and almost 50% have iron deficiency (ID) [5]. Both anemia and ID are associated with worst clinical outcomes in patients with HF. Whether these are the mediators of poor outcome or are just the bad prognostic markers, the debate is far from over. While treatment of ID has shown to produce symptomatic improvement in these patients, correction of anemia has failed to show any significant positive outcomes. In this article, we aim to review the existing data on management of anemia and ID in HF patients and discuss the future therapies under development.

## Main text

### Anemia in HF: prevalence

Prevalence of anemia in HF (hemoglobin (Hb) less than 12 gm/dL and 13 gm/dL in females and males respectively) has been reported between 17–70% depending on patients’ demographics, comorbidities, type of study and HF severity [6, 7]. The anemic patients with HF are found to be older and have more comorbidities like diabetes mellitus, chronic kidney disease (CKD) and have worse functional capacity with poorer quality of life. They tend to have lower blood pressure, more edema, higher requirement of diuretics [8–14].

### Anemia in HF: etiology

**Fig. 1:**
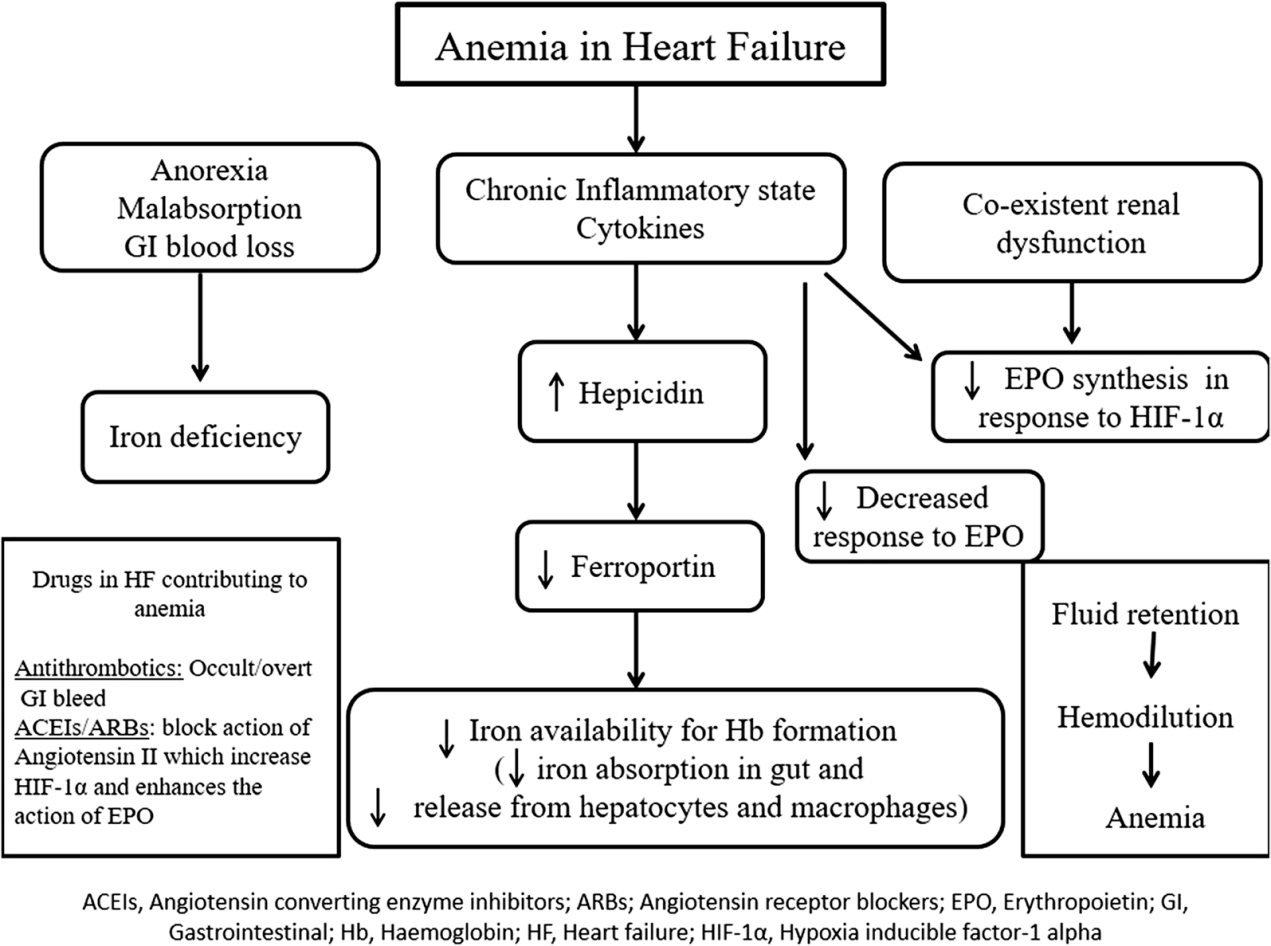
Etiology of anemia in heart failure

Anemia in HF is multifactorial. Fifty percent of patients with HF have ID; either they have depleted iron stores (low ferritin (less than 100 ug/dL) and low transferrin saturation (equal to or less than 20%) or they have functional iron deficiency in the form of normal iron stores (ferritin (100–300 ug/dL) and low transferrin saturation (equal to or less than 20%) [8, 9]. Transferrin saturation is obtained by dividing the serum iron by total iron binding capacity. The nutrient deficiency may occur either due to decreased intake or due to decreased absorption of iron in the gut. However, the deficiency of other nutrients like folic acid and vitamin B12 are less well described as contributing factors. HF is a complex inflammatory state which is associated with upgradation of inflammatory markers like interleukin-1, interleukin-6 and tumor necrosis factor and is usually associated with multiple comorbidities like CKD [13, 14]. The appropriate erythropoietin (EPO) response to anemia may be blunted due to kidney dysfunction. In addition, the cytokines produce a state of resistance to EPO. Though the upgradation of sympathetic and renin angiotensin system increases the production of EPO in kidneys, the drugs used in heart failure commonly blunts this response. Also the use of antithrombotics in patients with HF for various indications may lead to occult gastrointestinal blood loss leading to ID. Figure 1 summarizes the potential mechanisms of anemia in HF.

### Anemia in HF: pathophysiology

Anemia in HF decreases the delivery of oxygen to the tissues and aggravates the symptoms of dyspnea and fatigue with worsening quality of life. In a patient without HF, anemia produces a hyperdynamic state and compensates with increased heart rate and stroke volume. These reserves are limited in patients with HF, and hence, anemia can decompensate such hemodynamics. It may lead to adverse left ventricular modeling and demand supply mismatch. In a large meta-analysis, the crude mortality risk associated with anemia in HF was an odd ratio of 1.96 (95% confidence interval 1.74–2.21), and the adjusted hazard ratio was 1.46 (95% confidence interval 1.26–1.69) [15]. There has been data which shows that treatment of HF resolved anemia and brought the mortality risk to baseline; however, the treatment of anemia in HF has not been associated with consistent positive outcomes. On the other hand, correction of ID (overt and occult) in HF is associated with better quality of life and symptomatic improvement and is hence recommended to be evaluated for and treated, irrespective of Hb levels. However, the effect of treatment of ID in HF on hard outcomes is yet to be seen. Hence, the debate, whether the anemia is a marker of HF severity or it leads to adverse outcomes, is far from over.

### Anemia in HF: treatment

**Fig. 2:**
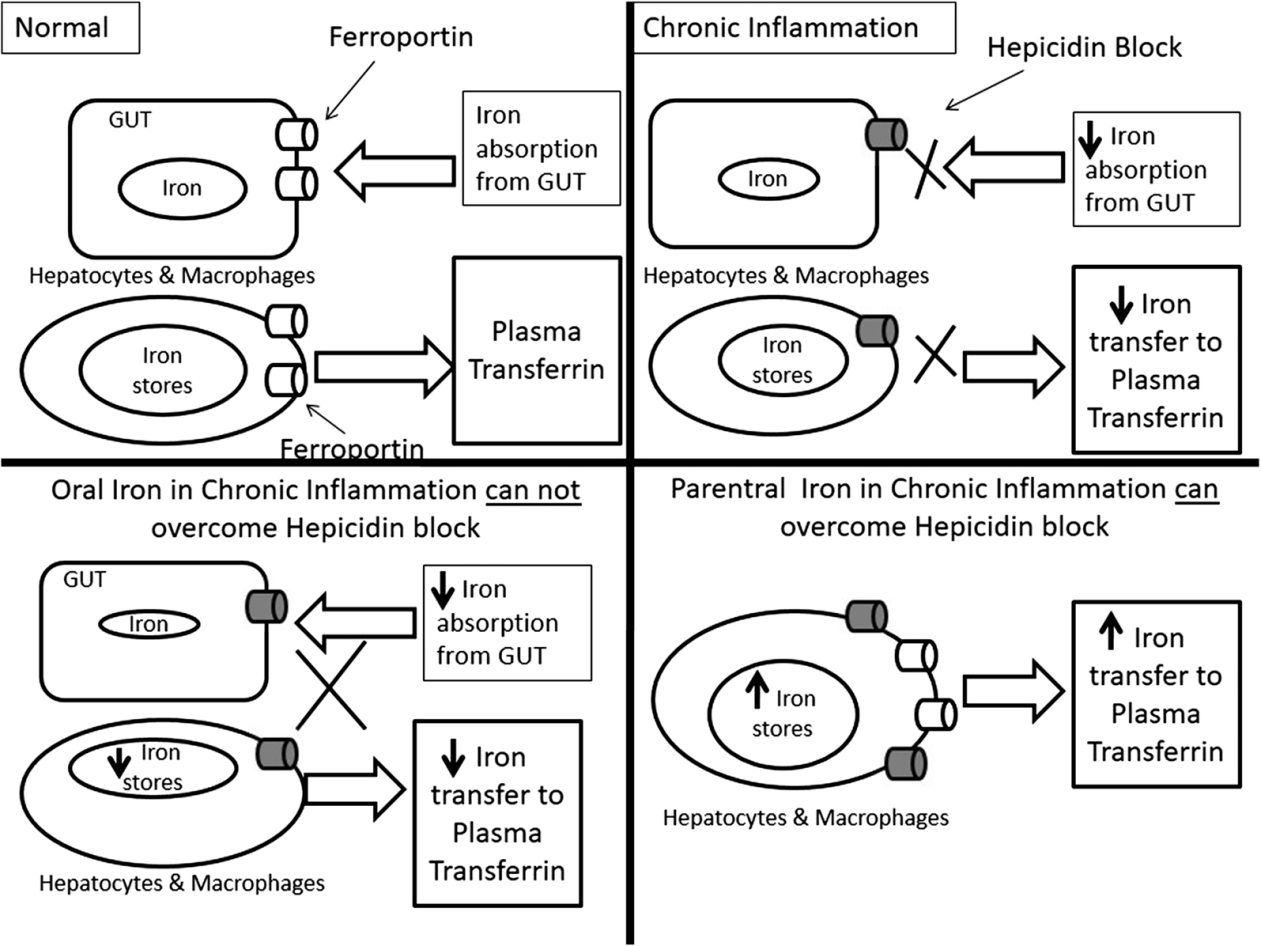
Role of parenteral iron in heart failure

1. Transfusion: In severe symptomatic anemia, a liberal transfusion strategy (trigger threshold of Hb 7–8 gm/dL) is recommended in patients with heart disease [16, 17]. Despite having some temporary benefits, the transfusion therapy can lead to volume overload and ischemic events in HF patients [18, 19], apart from other adverse events like hemolytic reactions, acute lung injury and infections.
2. Erythropoietin-Stimulating Agents (ESAs): Exogenous erythropoietin was studied in patients with HF in Reduction Of Events by Darbepoetin Alpha in Heart Failure (RED-HF) trial, where more than 2000 HF patients with ejection fraction equal to or less than 40% and anemia were randomized to receive Darbepoetin Alpha or placebo [20]. There was no difference in primary outcome (death or HF hospitalization) in two groups; however, there was significantly increased number of ischemic strokes and thromboembolic events in the ESA group. The reason for the observed outcome was heterogeneity of anemia in HF patients and a large proportion of HF patients already have high EPO levels, with resistance of bone marrow to its action [21]. Hence, EPO is not recommended to treat anemia in HF [22–24]. Even in patients with CKD, higher Hb targets with EPO are associated with worse cardiovascular outcomes.
3. Iron Therapy: Parenteral iron therapy, in patients with HF with reduced ejection fraction and iron deficiency, irrespective of Hb levels, has shown to improve New York Heart Association functional class, quality of life and exercise capacity [25–28]. The effect of parenteral iron therapy on hard outcomes is yet to be ascertained. Most of the studies have taken a ferritin cutoff of equal to or less than 100 ug/dL or ferritin of 100–300 ug/dL and transferring saturation of equal to or less than 20%. Recent studies have found that transferrin saturation, rather than ferritin levels, reflects the iron stores and availability more accurately, and hence should be used to guide the parenteral iron therapy [29, 30]. The most studied parenteral iron formulation is ferric carboxymaltose. Both the European and American guidelines recommend that iron deficiency should be ruled out in all HF patients, irrespective of Hb levels [17, 22–24]. Parenteral iron therapy is recommended in patients found to have iron deficiency. Oral iron therapy has been studied in the trial, IRONOUT (Oral Iron Repletion Effects On Oxygen Uptake in Heart Failure) [31]. However, in 16 weeks, the 300 mg of oral polysaccharide iron produced only marginal increase in ferritin and transferrin saturation, without any significant increase in exercise capacity or effect on N-terminal pro-B-type natriuretic peptide. Explanation for the observed effect is that due to inflammatory state of HF, the hepcidin levels are high which degrade the iron exporter ferroportion, thus blocking the iron absorption from the gut and the iron release from macrophages. This block is overcome by high concentration of intracellular iron after parenteral therapy. Figure 2 illustrates the mechanism of action of parenteral iron in chronic inflammatory state like HF. Table 1 summarizes the studies evaluating role of blood transfusion, ESA and iron in HF [32–35].
4. New therapies: The new therapies which are being evaluated for management of anemia in HF include the molecules which target hepcidin, hypoxia pathway and the EPO receptor.
5. Hepcidin: It can be antagonized by reducing its production, neutralizing it or preventing the hepcidin–ferroportion interaction. This shall increase the absorption of iron from the gut and increase the bioavailability of iron for erythropoiesis. A fully humanized monoclonal antibody against hepcidin (LY2787106) and a hepcidin-binding agent, the Spiegelmer lexaptepid (NOX-H94) have shown promising results in phase 1 trials [36, 37].
6. EPO receptor targeting: These include mimetic peptides, gene therapy, fusion proteins, receptor antibodies and active receptor ligand traps. These activin traps bind a large number of transforming growth factor β family ligands and inhibit their signaling. The proposed mechanisms of action include making erythropoiesis more efficient by reducing the number of growth differentiation factor-11-positive cells [38] or by increased expression of angiotensin II [39]. Since the later in not desirable in HF patients, these activin traps have not been studied in HF patients with anemia.
7. Hypoxia-Inducible Factor (HIF) Stabilizers: In low oxygen conditions, HIF induces transcription of multiple genes including EPO [40]. One of the HIF stabilizers, roxadustat (FG-4592), has shown to increase EPO and Hb levels and decrease the hepcidin in CKD patients [41].

**Table 1:** Studies evaluating role of blood transfusion, erythropoietin-stimulating agents and iron in heart failure

Trial/study	Finding	Recommendation/practice
*Blood transfusion (BT)*		
Hebert PC et al. Retrospective and Prospective Cohort [32] *n* = 4470 (critically ill patients)	Hb < 9.5gm/ dL associated with increased mortality in cardiac patients BT in anemic patients with cardiac disease and APACHE II score > 20 associated with improved survival	Transfusion threshold Hematocrit < 30% in cardiovascular disease (Based on expert opinion) [11] Transfusion for severe and symptomatic anemia in HF [17]
Hebert PC et al. Multicenter, Randomized Controlled Trial [33] *n* = 838 critically ill euvolemic patients Liberal BT strategy (Hb < 9 gm/dL) versus restrictive BT strategy (Hb < 7 gm/dL) strategy	Restrictive BT strategy as effective as liberal (perhaps superior) except in acute coronary syndrome patients	
Hebert PC et al. Randomized Controlled Trial [34] *n* = 357 critically ill patients with cardiovascular disease Liberal BT strategy (Hb < 10 gm/dL) [*n* = 197] versus restrictive BT strategy (Hb < 7 gm/dL) [*n* = 160]strategy	Restrictive BT strategy as effective as liberal (perhaps superior) except in acute coronary syndrome patients	
Garty et al. Prospective Cohort study ( Hospital based HF survey in Israel (HFSIS) [18] *n* = 4,102 (CHF [* n* = 1767] and ADHF [*n* = 2335]	After propensity score analysis, blood transfusion was associated with lower short term mortality; however, there is no difference in long term mortality	
*Erythropoietin-stimulating agents*		
STAMINA HeFT trial. Randomized Controlled Trial [35] *n* = 319 patients (follow-up—53 weeks) Inclusion criteria: LVEF ≤ 40%, Hb 9 -12.5 g/dl Target Hb: 13 to 15 g/dl Intervention: Darbepoetin Alfa [*n* = 162] versus placebo [*n* = 157]	No significant difference in exercise duration, NYHA class or QoL Nonsignificant trend observed toward a lower risk of all-cause mortality or first HF hospitalization in darbepoetin alfa-treated group Adverse events similar in both arms	Erythropoietin-stimulating agents are not recommended to be used for treatment of anemia in HF [17, 22–24]
RED-HF trial. Double blind Randomized Controlled Trial [20] *n* = 2278 patients (follow-up—28 months) Inclusion Criteria: LVEF ≤ 35%, Hb 9–12 g/dl Target Hb: 13 to 14.5 g/dl Intervention: Darbepoetin alfa [*n* = 1136] versus placebo [*n* = 1142]	No difference in primary outcome (all-cause death or first hospitalization for worsening HF Significant increase in incidence of ischemic cerebrovascular accident and thromboembolic events with Darbepoetin alfa	
*Parenteral iron*		
FAIR-HF. Multicenter, Double blind Randomized Controlled trial [25] *n* = 459 [follow-up—24 weeks] Inclusion criteria: LVEF < 40% (NYHA class II) or < 45% (NYHA III) with ID (ferritin < 100 ng/mL or 100–300 ng/mL if TSAT < 20%) and anemia (Hb 9.5–12 gm/dl) or without anemia (Hb 12.0–13.5 gm/dl) Intervention: Parenteral iron-FCM [*n* = 304] versus placebo [*n* = 155]	Significant improvement in NYHA class, 6MWT, QoL and patient global assessment	ESC/ACC guidelines: Parenteral iron (preferable FCM or non-dextran iron) for symptomatic HF patients (NYHA II and III) with ID (ferritin < 100 ug/dl or ferritin between 100–299 ug/dL and TSAT < 20%) to improve symptoms and QoL [13–17]
CONFIRM-HF. Multicenter, Double blind Randomized Controlled trial [26] *n* = 304 [follow-up—52 weeks] Inclusion criteria: LVEF ≤ 45%, symptomatic HF with elevated natriuretic peptides and ID (ferritin < 100 ng/mL or 100–300 ng/mL if TSAT < 20%) Intervention: Parenteral iron—FCM [*n* = 152] versus placebo [*n* = 152]	Significant Improvement in NYHA class, 6MWT QoL and patient global assessment Significant reduction in the risk of hospitalizations for worsening HF	
EFFECT-HF. Randomized Controlled Trial [27] *n* = 172 [follow-up—24 weeks] Inclusion criteria: LVEF ≤ 45%, NYHA class II/III despite optimal medical therapy for HF ≥ 4 weeks Intervention: Parenteral iron-FCM [*n* = 86] versus placebo [*n* = 86]	Significant increase in Peak oxygen consumption Significant improvement in NYHA class and patient global assessment Significant increase in iron stores	
AFFIRM-AHF. Multicenter, Double blind Randomized Controlled trial [28] *n* = 1132 [follow-up—52 weeks] Inclusion criteria: LVEF < 50%, ADHF with concomitant ID (ferritin < 100 ng/mL or 100–300 ng/mL if TSAT < 20%) Intervention: Parenteral iron- FCM [*n* = 558] versus placebo [*n* = 550] [Intervention after stabilization, before discharge]	Significant decrease in HF related hospitalizations No difference in cardiovascular deaths Parenteral iron safe	
*Ongoing trials with parenteral iron in HF*		
FAIR-HF2. Multicenter, Double blind Randomized Controlled trial [ClinicalTrials.gov Identifier: NCT03036462] Estimated *n* = 1200 [Expected follow-up—52 weeks] Inclusion criteria: Systolic HF with documented ID Intervention: Parenteral iron- FCM versus placebo	Primary outcome: Combined rate of recurrent cardiovascular hospitalizations and of cardiovascular death [ongoing]	
HEART-FID. Multicenter, Double blind Randomized Controlled trial [ClinicalTrials.gov Identifier: NCT03037931] Estimated *n* = 3014 [Expected follow-up—52 weeks] Inclusion criteria: LVEF ≤ 40%, NYHA II, III with documented ID Intervention: Parenteral iron- FCM versus placebo	Primary outcomes: Incidence of death at 1 year Incidence of hospitalization for HF at 1 year Change in 6MWT distance at 6 months [ongoing]	
*Oral iron*		
IRONOUT HF. Randomized Controlled trial [31] *n* = 225 [follow-up—16 weeks] Inclusion criteria: NYHA II-IV, LVEF ≤ 40% and ID (ferritin 15–100 ng/mL or between 100–299 ng/mL with a TSAT < 20%) and Hb: 9–15 g/dL (men) and 9–13.5 g/dL (women) Intervention: oral iron polysaccharide (150 mg twice a day) [*n* = 111] versus placebo [*n* = 114]	No significant difference between peak oxygen consumption between two groups No significant difference in exercise capacity, 6MWT, NT-pro-BNP and KCCQ Clinical Summary score	Oral iron: not enough evidence

ACC, American College of Cardiology; ADHF, acute decompensated heart failure; APACHE, Acute Physiology and Chronic Health Evaluation; BT, blood transfusion; ESC, European Society of Cardiology; FCM, ferric carboxymaltose; Hb; hemoglobin; HF, heart failure; ID, iron deficiency; KCCQ, Kansas City Cardiomyopathy Questionnaire; LVEF, left ventricular ejection fraction; 6MWT; 6-min walk test; NT-pro BNP, N-terminal pro-B-type natriuretic peptide; NYHA, New York Heart Association; TSAT, transferrin saturation; QoL, quality of life

### Anemia in HF: current guidelines

The current guidelines recognize that anemia is an important prognostic marker in HF patients and lay stress on evaluating the etiology of same, though most of the times no specific cause is found [17, 22–24]. A special emphasis is laid on correction of iron deficiency in HF with parenteral iron, irrespective of the Hb levels, to improve the functional status.

## Conclusion

Despite all the research, anemia in HF remains an enigma. Affecting almost one-third of HF patients, anemia is associated with bad outcomes. However, the treatment of anemia and rise in Hb levels have not been consistently linked to better prognosis. Though parenteral iron improves the functional capacity in iron deficient HF patients, the effects are independent of Hb levels; and also the evidence on hard clinical outcomes is yet to be ascertained.

## Data Availability

Not applicable.

## Abbreviations

CKD: Chronic kidney disease
EPO: Erythropoietin
ESAs: Erythropoietin-stimulating agents
Hb: Hemoglobin
HF: Heart failure
HIF: Hypoxia-inducible factor
ID: Iron deficiency

## References

[1] Ziaeian B, Fonarow GC (2016). Epidemiology and aetiology of heart failure. Nat Rev Cardiol.

[2] Mazurek JA, Jessup M (2017). Understanding Heart Failure. Heart Fail Clin.

[3] Yancy CW, Jessup M, Bozkurt B, Butler J, Casey DE Jr, Drazner MH, et al; American College of Cardiology Foundation/American Heart Association Task Force on Practice Guidelines (2013) 2013 ACCF/AHA guideline for the management of heart failure: a report of the American College of Cardiology Foundation/American Heart Association Task Force on practice guidelines. Circulation 128(16):e240–327. 10.1161/CIR.0b013e31829e8776 23741058

[4] Ponikowski P, Anker SD, AlHabib KF, Cowie MR, Force TL, Hu S (2014). eart failure: preventing disease and death worldwide. ESC Heart Fail.

[5] van Veldhuisen DJ, Anker SD, Ponikowski P, Macdougall IC (2011). Anemia and iron deficiency in heart failure: mechanisms and therapeutic approaches. Nat Rev Cardiol.

[6] Waldum B, Westheim AS, Sandvik L, Flonæs B, Grundtvig M, Gullestad L (2012). Baseline anemia is not a predictor of all-cause mortality in outpatients with advanced heart failure or severe renal dysfunction. Results from the Norwegian Heart Failure Registry. J Am Coll Cardiol.

[7] Grote Beverborg N, van Veldhuisen DJ, van der Meer P (2018). Anemia in Heart Failure: Still Relevant?. JACC Heart Fail.

[8] Anand IS, Gupta P (2018). Anemia and iron deficiency in heart failure. Circulation.

[9] Anand IS (2008). Anemia and chronic heart failure implications and treatment options. J Am Coll Cardiol.

[10] Ezekowitz JA, McAlister FA, Armstrong PW (2003). Anemia is common in heart failure and is associated with poor outcomes: insights from a cohort of 12065 patients with new-onset heart failure. Circulation.

[11] Goodnough LT, Schrier SL (2014). Evaluation and management of anemia in the elderly. Am J Hematol.

[12] Anand IS, Kuskowski MA, Rector TS, Florea VG, Glazer RD, Hester A (2005). Anemia and change in hemoglobin over time related to mortality and morbidity in patients with chronic heart failure: results from Val-HeFT. Circulation.

[13] Opasich C, Cazzola M, Scelsi L, De Feo S, Bosimini E, Lagioia R (2005). Blunted erythropoietin production and defective iron supply for erythropoiesis as major causes of anaemia in patients with chronic heart failure. Eur Heart J.

[14] Anand IS, Rector T, Deswal A, Iverson E, Anderson S, Mann D (2006). Relationship between proinflammatory cytokines and anemia in heart failure. Eur Heart J.

[15] Groenveld HF, Januzzi JL, Damman K, van Wijngaarden J, Hillege HL, van Veldhuisen DJ (2008). Anemia and mortality in heart failure patients a systematic review and meta-analysis. J Am Coll Cardiol.

[16] Qaseem A, Humphrey LL, Fitterman N, Starkey M, Shekelle P (2013). Clinical Guidelines Committee of the American College of Physicians. Treatment of anemia in patients with heart disease: a clinical practice guideline from the American College of Physicians. Ann Intern Med.

[17] Hollenberg SM, Warner Stevenson L, Ahmad T, Amin VJ, Bozkurt B, Butler J (2019). 2019 ACC expert consensus decision pathway on risk assessment, management, and clinical trajectory of patients hospitalized with heart failure: a report of the American College of Cardiology Solution Set Oversight Committee. J Am Coll Cardiol.

[18] Garty M, Cohen E, Zuchenko A (2009). Blood transfusion for acute decompensated heart failure—friend or foe?. Am Heart J.

[19] Kao DP, Kreso E, Fonarow GC, Krantz MJ (2011). Characteristics and outcomes among heart failure patients with anemia and renal insufficiency with and without blood transfusions (public discharge data from California 2000–2006). Am J Cardiol.

[20] Swedberg K, Young JB, Anand IS, Cheng S, Desai AS, Diaz R (2013). RED-HF committees; RED-HF investigators. Treatment of anemia with darbepoetin alfa in systolic heart failure. N Engl J Med.

[21] van der Meer P, Voors AA, Lipsic E, Smilde TDJ, van Gilst WH, van Veldhuisen DJ (2004). Prognostic value of plasma erythropoietin on mortality in patients with chronic heart failure. J Am Coll Cardiol.

[22] Yancy CW, Jessup M, Bozkurt B, Butler J, Casey DE, Colvin MM (2017). ACCF/ACC/HFSA focused update on new pharmacological therapy for heart failure: an update of the 2013 ACCF/AHA guidelines for the management of heart failure: a report of the American College of Cardiology Foundation/American Heart Association Task Force on Practice Guidelines and the Heart Failure Society of America. J Am Coll Cardiol.

[23] Ponikowski P, Voors AA, Anker SD, Bueno H, Cleland JGF, Coats AJS et al. ESC Scientific Document Group (2016) 2016 ESC Guidelines for the diagnosis and treatment of acute and chronic heart failure: The Task Force for the diagnosis and treatment of acute and chronic heart failure of the European Society of Cardiology (ESC) Developed with the special contribution of the Heart Failure Association (HFA) of the ESC. Eur Heart J 37(27):2129–2200. 10.1093/eurheartj/ehw128 27206819

[24] Seferovic PM, Ponikowski P, Anker SD, Bauersachs J, Chioncel O, Cleland JGF (2019). Clinical practice update on heart failure 2019: pharmacotherapy, procedures, devices and patient management. An expert consensus meeting report of the Heart Failure Association of the European Society of Cardiology. Eur J Heart Fail.

[25] Anker SD, Comin-Colet JC, Filippatos G, Willenheimer R, Dickstein K, Drexler H, FAIR-HF Investigators et al (2009) Ferric carboxymaltose in patients with heart failure and iron deficiency. N Engl J Med 361:2436–2348. 10.1056/NEJMoa0908355 19920054

[26] Ponikowski P, van Veldhuisen DJ, Comin-Colet J, Ertl G, Komajda M, Mareev V, CONFIRM-HF Investigators et al (2015) Beneficial effects of long-term intravenous iron therapy with ferric carboxymaltose in patients with symptomatic heart failure and iron deficiency†. Eur Heart J 36(11):657–668. 10.1093/eurheartj/ehu385 PMC4359359 25176939

[27] van Veldhuisen DJ, Ponikowski P, van der Meer P, Metra M, Böhm M, Doletsky A, EFFECT-HF Investigators et al (2017) Effect of ferric carboxymaltose on exercise capacity in patients with chronic heart failure and iron deficiency. Circulation 136(15):1374–1383 10.1161/CIRCULATIONAHA.117.027497 PMC5642327 28701470

[28] Ponikowski P, Kirwan BA, Anker SD, McDonagh T, Dorobantu M, Drozdz J, AFFIRM-AHF investigators, et al (2020) Ferric carboxymaltose for iron deficiency at discharge after acute heart failure: a multicentre, double-blind, randomised, controlled trial. Lancet 396(10266):1895–1904. 10.1016/S0140-6736(20)32339-4 33197395

[29] Grote Beverborg N, van der Meer P (2017). Definition of iron deficiency based on the gold standard of bone marrow iron staining and treatment effect of ferric carboxymaltose in heart failure patients (abstr). Eur J Heart Fail.

[30] Anker SD, Kirwan BA, van Veldhuisen DJ, Filippatos G, Comin-Colet J, Ruschitzka F (2018). Effects of ferric carboxymaltose on hospitalisations and mortality rates in iron-deficient heart failure patients: an individual patient data meta-analysis. Eur J Heart Fail.

[31] Lewis GD, Semigran MJ, Givertz MM, Malhotra R, Anstrom KJ, Hernandez AF (2016). Oral iron therapy for heart failure with reduced ejection fraction: design and rationale for oral iron repletion effects on oxygen uptake in heart failure. Circ Heart Fail.

[32] Hébert PC, Wells G, Tweeddale M, Martin C, Marshall J, Pham B (1997). Does transfusion practice affect mortality in critically ill patients? Transfusion Requirements in Critical Care (TRICC) Investigators and the Canadian Critical Care Trials Group. Am J Respir Crit Care Med.

[33] Hebert PC, Yetisir E, Martin C (2001). Transfusion Requirements in Critical Care Investigators for the Canadian Critical Care Trials Group. Is a low transfusion threshold safe in critically ill patients with cardiovascular diseases?. Crit Care Med.

[34] Hebert PC, Wells G, Blajchman MA, Marshall J, Martin C, Pagliarello G (1999). A multicenter, randomized, controlled clinical trial of transfusion requirements in critical care. Transfusion requirements in critical care (TRICC)investigators. Canadian critical care trials group. N Engl J Med.

[35] Ghali JK, Anand IS, Abraham WT, Fonarow GC, Greenberg B, Krum H, et al. Study of Anemia in Heart Failure Trial (STAMINA-HeFT) Group (2008) Randomized double-blind trial of darbepoetin alfa in patients with symptomatic heart failure and anemia. Circulation 117(4):526–535. 10.1161/CIRCULATIONAHA.107.698514 18195176

[36] Vadhan-Raj S, Abonour R, Goldman JW, Smith DA, Slapak CA, Ilaria RL (2017). A first-in-human phase 1 study of a hepcidin monoclonal antibody, LY2787106, in cancer-associated anemia. J Hematol Oncol.

[37] van Eijk LT, John AS, Schwoebel F, Summo L, Vauléon S, Zöllner S (2014). Effect of the antihepcidin Spiegelmer lexaptepid on inflammation-induced decrease in serum iron in humans. Blood.

[38] Paulson RF (2014). Targeting a new regulator of erythropoiesis to alleviate anemia. Nat Med.

[39] Iancu-Rubin C, Mosoyan G, Wang J, Kraus T, Sung V, Hoffman R (2013). Stromal cell-mediated inhibition of erythropoiesis can be attenuated by Sotatercept (ACE-011), an activin receptor type II ligand trap. Exp Hematol.

[40] Semenza GL (1998). Hypoxia-inducible factor 1: master regulator of O2 homeostasis. Curr Opin Genet Dev.

[41] Chen N, Qian J, Chen J, Yu X, Mei C, Hao C (2017). Phase 2 studies of oral hypoxia-inducible factor prolyl hydroxylase inhibitor FG-4592 for treatment of anemia in China. Nephrol Dial Transplant.

